# COMPRESSIVE DATA STORAGE FOR LONG-TERM EEG: VALIDATION BY VISUAL ANALYSIS

**DOI:** 10.1016/j.cnp.2025.07.005

**Published:** 2025-08-05

**Authors:** Giridhar P. Kalamangalam, Subeikshanan Venkatesan, Maria-Jose Bruzzone, Yue Wang, Carolina B. Maciel, Sotiris Mitropanopoulos, Jean Cibula, Kajal Patel, Abbas Babajani-Feremi

**Affiliations:** a Department of Neurology, University of Florida, Gainesville, FL, USA; b Wilder Center for Epilepsy Research, University of Florida, Gainesville, FL, USA; c Department of Neurology, Mayo Clinic, Scottsdale, AZ, USA; d Department of Neurology, Cleveland Clinic, Cleveland, OH, USA; e MEG Laboratory, Norman Fixel Institute for Neurological Diseases at UF Health, Gainesville, FL, USA

**Keywords:** Data science, Critical care monitoring, Singular value decomposition, Discrete cosine transform

## Abstract

**Objectives:**

Long-term EEG monitoring (LTM) in acute neurology generates massive data volumes. We investigated whether data-analytic techniques could reduce LTM data size yet conserve their visual diagnostic features.

**Methods:**

LTM exemplars from 50 patients underwent singular value decomposition (SVD). High-variance SVD components were transformed using discrete cosine transform (DCT), and significant elements run-length encoded. Two regimes were tested: (I) SVD and DCT compression ratio (CR) of 1.7 and 12, and (II) CR of 3.7 and 5.7; each achieved an overall CR of ≈20. Compressed data were reconstructed alongside uncompressed originals, to create a total of 200 recordings that were scored by two blinded reviewers. Scores of original and reconstructed data were statistically analyzed.

**Results:**

Score differences between original recordings were smaller than comparisons involving reconstructions using the first regime but did not differ significantly from reconstructions using the second regime.

**Conclusions:**

Raw LTM EEG has sufficient redundancy to undergo extreme (20-fold) data compression without compromising visual diagnostic information. A balanced mix of SVD and DCT appears to be a suitable data-analytic pipeline for achieving such compression.

**Significance:**

Dimension reduction is a significant goal in managing big biomedical data. Our results suggest a pathway for archival of meaningful representations of entire LTM datasets. The latent space suggests new lines of data-scientific inquiry of the EEG in acute neurological illness.

## Highlights

• Long-term EEG monitoring (LTM) accrues massive data volumes that are challenging to permanently archive in their entirety.
• Analytic techniques can achieve a 20-fold compression of LTM data size without compromising visually diagnostic features.
• The latent space may suggest new scientific questions in the EEG of acute neurological illness.

## 1 Introduction

Long-term EEG monitoring (LTM) is increasingly used in hospitalized patients (Hill et al., 2019) and accrues substantial data volumes. The opportunities posed by these significant LTM datasets are relevant to wider topical discussions on ‘big’ data in epilepsy (Baldassano et al., 2019, Lhatoo et al., 2020) and neuroscience (Landhuis, 2017, Thompson et al., 2020). For individual institutions, an important – if mundane – challenge is storage capacity and costs associated with permanent archival of these data. Consequently, most institutions retain only ‘prunes’ (or ‘clips’); that is, they delete all raw data except the fragments considered to be diagnostic significance. Such large-scale deletion defeats the process of big data and precludes post-hoc analysis, retrospective research, and clinical audit. We inquired whether raw EEG could instead be ‘compressed’ – i.e., dimension-reduced to compact representations – for storage in a manner that preserved clinical utility.

## 2 Methods

### 2.1 Data acquisition

We prospectively obtained one-hour segments of artifact-free scalp EEG data (1–70 Hz passband at 256 Hz; XLTEK®, Natus Medical Inc., Middleton, WI) from 50 LTM patients. The range of clinical conditions represented comprised those in common LTM practice – encephalopathy of uncertain cause, seizures or suspected seizures, traumatic brain injury, intracranial hemorrhage and cerebrovascular disease. Candidate recordings were flagged by rotating service physicians over a three-month period (GPK, JEC, SM) as they encountered typical examples of EEGs showing background slowing, background asymmetry, seizures, and rhythmic and periodic patterns. EEG segments were moved from clinical servers to research storage in European Data format (EDF) for offline analysis.

### 2.2 Data transformation

The data were imported into MATLAB® (The Mathworks, Inc., Natick, MA) in referential format and segmented into 10-s epochs. Each epoch of scalp EEG was thus a 2-D m×n matrix, with m being the list of 21 head locations in the International 10–20 system and n the successive data points (256 × 10 = 2560 for 256 Hz sampling frequency). Each epoch was dimension-reduced in the five-step sequence outlined below. In the sixth and final step, the reduced data were reconstructed and visually reviewed. Formal details on the methods appear in the Appendix.

(i) Singular value decomposition (SVD): SVD (Strang, 2009) views each data channel as a vector in a high-dimensional space, and projects the vector set in an orthogonal sequence onto the directions of maximum variance. The projection of each data epoch yielded 21 singular vectors, each of length 2560, and ordered by their singular value magnitudes. Data reduction was achieved by retaining only the few singular vectors associated with the largest singular values. We experimented with retaining between 5 and 15 of the full set of 21 singular vectors (below).
(ii) Discrete Cosine Transform (DCT): Each of the retained singular vectors was next subjected to DCT. The DCT is an algorithm that projects a data vector onto a set of cosine functions of different frequencies (Ahmed and Rao, 1974). We adopted the commonly-used DCT-2 formulation (Strang, 1999) here. Each DCT yielded a vector of coefficients of the same size as the singular vector. Again, only the fraction comprising the largest DCT coefficients were retained. We experimented with retaining between 5 and 20 % of the full set of DCT coefficients (below).
(iii) Two-byte quantization: The retained DCT coefficients were then quantized to two bytes in preparation for run-length encoding below.
(iv) Run-length Encoding (RLE): The quantized coefficients were subjected to RLE, a technique that compresses sequences of zeros by representing them with a single zero value followed by a count (Salomon, 2007), utilizing two bytes for the quantization bit number.
(v) Iteration for DCT coefficient retention: While thresholding DCT coefficients allows precise control over the number of retained coefficients, the actual compression for RLE depends on the pattern of zeros in the sequence of coefficients. To achieve an exact desired compression, we used an iterative algorithm that retained a variable number of the largest coefficients, setting the rest to zero. The coefficients were quantized and then compressed using RLE. The overall compression ratio (CR; achieved by SVD and DCT together) was calculated for a given number of DCT coefficients, and the number of retained coefficients adjusted by a Newton-Raphson iteration until the achieved CR matched a desired value.
(vi) Reconstruction: Inverse RLE expanded the compressed data into the original sequence of quantized coefficients, which were then inverse quantized to restore their original range and precision. The inverse DCT was then applied to reconstruct the time courses of the original singular vectors. The reconstructed singular vectors and their corresponding singular values were used to recover the individual 10-s epochs of EEG data, which were concatenated into one-hour long segments in EDF format for visual review in Persyst® (Persyst, Inc., Solana Beach, CA).

We evaluated two ways of achieving our target CR = 20. Regime I had individual SVD and DCT CRs of 1.7 and 12 respectively (COMP1; 1.7 × 12 ≈ 20) and Regime II had CRs of SVD and DCT of 3.7 and 5.7, respectively (COMP2; 3.7 × 5.7 ≈ 20).

### 2.3 Visual review and scoring

COMP1 and COMP2, along with two copies of the original EEG (ORIG1 and ORIG2) for each patient were randomized into a list of 200 records (4 records/subject × 50 subjects). All records were reviewed in sequence by two board certified electroencephalographers (M−JB, YW), who were blind to the initial data selection process.

Records were evaluated on 45 diagnostic features, grouped under three major categories: (I) background, (II) focal abnormalities, and (III) hyperexcitability features. The overall scheme of evaluation mirrored the 2021 ACNS standardized critical care EEG nomenclature (Hirsch et al., 2021), with each primary diagnostic feature specified by increasingly detailed attributes. Thus, each major category had sub-categories A, B, C, etc., that were qualified in a branching structure (Supplementary Fig. 1a–c). For background, the branches comprised symmetry (A), longitudinal organization (B), continuity (C) and the predominant frequency (D). Similarly, focal abnormalities were sub-categorized by slowing (A) and attenuation (B). Features of hyperexcitability were sub-categorized by sporadic epileptiform discharges (A), rhythmic and periodic patterns (B) and discrete evolving seizures (C). Sub-categories branched further into specific qualifiers that specified the further attributes of the parent diagnostic feature. Reviewers were asked to score each record across the entire template. Some features, such as seizures, required a binary response (i.e., 1 – present and 0 – absent). Others required responses on a nominal scale (e.g., localization of the seizure onset: 1 – right, 2 – left, 3 – generalized, or 4 – unclear), or solicited a numerical response (e.g. burst suppression duration). The final ‘score’ of a record was thus a mixture of binary, nominal, and numerical responses.

The final total of 200 scores (per reviewer) comprised four scores per individual EEG: the original was scored twice (ORIG1, ORIG2), and the reconstructed COMP1 and COMP2 versions scored once each. We next quantified the degree of discordance between scores, for each of the five combinations per EEG (ORIG1 – ORIG2 [O11], ORIG1 – COMP1 [D11], ORIG1 – COMP2 [D12], ORIG2 – COMP1[D21], ORIG2 – COMP2 [D22]). Penalties were introduced for disagreements in the response accorded to diagnostic features. As shown in Supplementary Fig. 1a–c (vertical double-headed arrows), the penalty scheme was hierarchical: ‘major’ (upstream) disagreements were penalized more heavily than ‘minor’ (downstream)) disagreements. For instance, a penalty of 14 was accorded to the disagreement of a seizure being marked as ‘present’ on one EEG version but ‘absent’ on another. However, disagreement on just the lobar location of a seizure only carried a penalty of 1. Similarly, disagreement on the presence or absence of focal slowing was accorded a penalty of 4, but disagreement on its lateralization carried the lower penalty of 3. Penalties were set up in such a way that within individual major categories, cumulative disagreement penalties for the qualifying features could not exceed the major disagreement penalty. Also, the penalty-assigning process was stopped at the first instance of a disagreement along the left-to-right hierarchy. This was done to avoid repeated penalization of the same attribute further downstream. For nominal or binary responses, disagreement scoring was straightforward. For numerical responses, we used a threshold of a 20 % difference to impose penalties.

If two scores agreed on every attribute, the omnibus penalty was 0; if they disagreed on every major attribute possible, the omnibus penalty was the maximum of 60.

### 2.4 Statistical analysis

Disagreement scores were reported as medians and interquartile ranges (IQRs). Baseline intra-reviewer variability was taken as the metric against which all other comparisons were made. That is, the null hypothesis was that the median disagreement by a reviewer between two copies of the original EEG (O11) was not statistically smaller than the same reviewer’s median disagreement between the original and its reconstructed versions (D11, D12, D21 or D22). The Wilcoxon signed rank test was used to compare the difference between pair-wise disagreement scores (IBM SPSS v29).

The study was approved by the Institutional Review Board of the University of Florida.

## 3 Results

For the first reviewer, median O11 was 4.75 and IQR was 9.63. Results for the original versus reconstructed EEG comparisons were: D11 median = 7.65 (IQR = 13); D12 median = 4.8 (IQR = 11.6); D21 median = 6.4 (IQR = 12.1); and D22 median = 6.9 (IQR = 9.4). O11 was significantly smaller than D11 (*p* < 0.003) and was smaller than D21 with borderline significance (*p* < 0.08). O11 was not significantly smaller than D12 (*p* > 0.14) or D22 (*p* > 0.4).

For the second reviewer, median O11 was 4.9 and IQR was 10.73. Results for the original versus reconstructed EEG comparisons were: D11 median = 7.4 (IQR = 14.7); D12 median = 5.5 (IQR = 13.2); D21 median = 6.8 (IQR = 11.6); and D22 median = 6.4 (IQR = 12.4). O11 was significantly smaller than D11 (*p* < 0.05) and D21 with borderline significances (*p* < 0.06). O11 was not significantly smaller than D21 (*p* > 0.28) or D22 (*p* > 0.22).

## 4 Discussion

The ascendancy of continuous long-term EEG for brain monitoring in acute and critical care neurology (Hill et al., 2019) entails an equivalent cost in maintaining the acquired data. At our institution video-EEG stores currently exceed 1 PB and recurring costs of data stewardship exceed $0.25 M annually (L Caillouet & R Turner, personal communication). The trivial solution to these significant resource requirements is to delete raw data and retain only pruned segments (‘clips’). Though practiced widely, such deletion precludes fuller retrospective analysis and research and is antithetical to the current era of ‘big’ data. The ideal solution to this data storage-versus-cost conundrum is if the data were somehow compressed to occupy less space, without the compressive process degrading their diagnostic essence. In this work we used two classical data transformation techniques – singular value decomposition (SVD) (Stewart, 1993) and the discrete cosine transform (DCT) (Ahmed and Rao, 1974) – for dimension reduction. When reconstructed, the data showed no significant degradation of diagnostic information, as judged by conventional clinical review.

There is a sizeable engineering literature on EEG data compression (Shaw et al., 2018, Gurve et al., 2023, Lerogeron et al., 2023, Morabito et al., 2013, Lal et al., 2023, Al-Marridi et al., 2018, Cardenas-Barrera and Lorenzo-Ginori, 2004, Wongsawat et al., 2006, Fira et al., 2021, Khafaga et al., 2023, Mammone et al., 2018, Hejrati et al., 2017, Antoniol and Tonella, 1997, Hinrichs, 1991, Capurro et al., 2017, Daou and Labeau, 2012, Agarwal and Gotman, 2001, Srinivasan et al., 2013, Dauwels et al., 2013, Casson et al., 2007, Islam and Xing, 2021) going back to the arrival of digital EEG (Nuwer, 1990), though no work relevant to neurological disease that we are aware of. The techniques we use here – SVD, DCT and run-length encoding – are widely used, and their performance judged by numerical metrics such as root-mean-square (RMS) distortion for a given CR. As discussed below, such metrics may not be literally applicable to clinical diagnosis. Thus, though the engineering literature provides an extensive backdrop, our work here was clinically motivated and took a somewhat different approach.

**Fig. 1:**
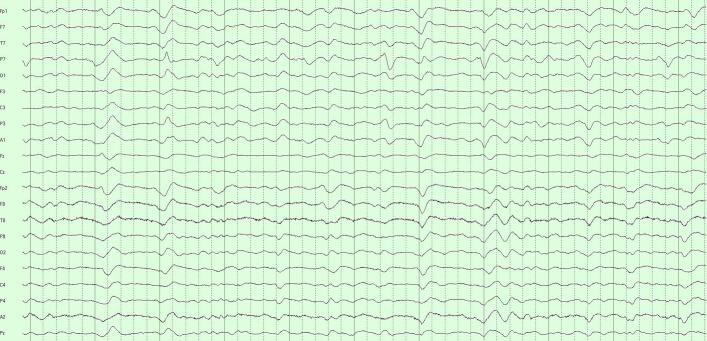

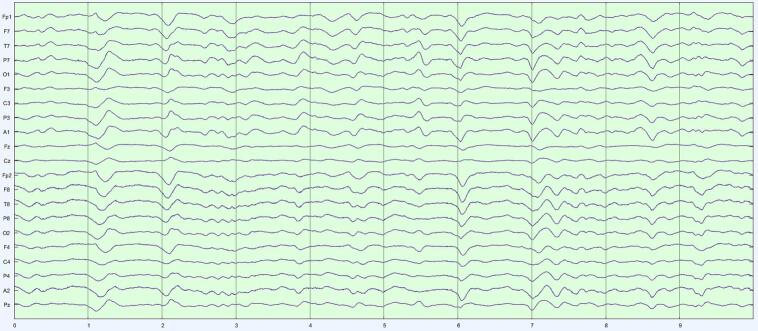

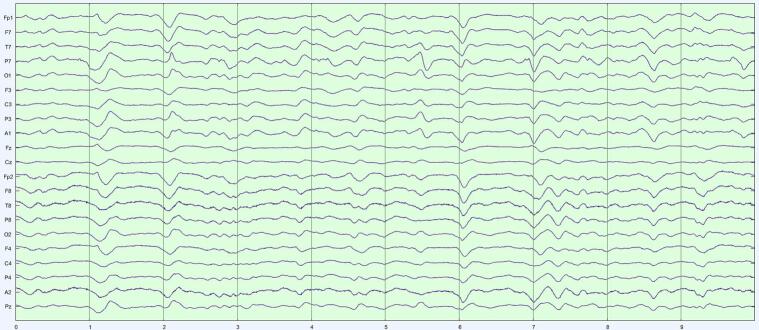
SVD reconstructions with progressively more components. a) Generalized slowing with some sharper waveforms seen on a 10 s EEG page in a patient with diffuse encephalopathy (referential montage, gain 7 μV/mm, passband 1–70 Hz). b) Reconstructed page with the first three SVD components. the overall generalized slow feature is captured, but faster and sharper features are not, as though the original EEG were low-pass filtered. c) Inclusion of five SVD components reconstructs the EEG more convincingly, with sharper components well seen.

We worked with LTM in acute neurological patients, rather from the epilepsy monitoring unit (EMU), for two reasons. First, the volume of LTM in acute neurology far exceeds data accruing from EMU in our institution. Compression is therefore most relevant to LTM in acute neurology. The second reason concerns the properties of the LTM data themselves. One common indication for LTM is to monitor encephalopathy in a neurologically altered patient (Hill et al., 2019). Encephalopathic EEG is characterized by ‘diffuse’ features, i.e., findings that are similar across multiple channels. Thus, though all EEG waveforms have spatial distributions (‘fields’), the fields of encephalopathy are especially broad. Viewed as data, diffuse EEG patterns are redundant: if similar patterns are seen in several channels, perhaps the content of a single (or a few) channel(s) can approximate *all* the data? In the terminology of linear algebra (Strang, 2009), the data matrix of diffuse slowing would be ‘low rank’: a subset of independent channels sufficiently encodes information of the entire data matrix. SVD is exactly a factorization that splits a data matrix into a sum of low-rank (rank-1) ingredients, and in order of importance. Our choice was just how many such ingredients to include in our approximation. Fig. 1a–c show SVD at work on a 10-s page of EEG that in a patient with generalized encephalopathy. The original data (Fig. 1a) are a broad mix of delta and theta with some sharper components. Fig. 1b is the reconstructed EEG from three SVD components. The diffuse nature of the rhythms is recovered; the EEG looks smoother due to lack of inclusion of faster frequencies. Fig. 1c represents the reconstruction of a five-component SVD. This page looks more like the original due to the inclusion of more information from the original, and acceptably represents the sharp components. For this 21-channel example, three channels (Fig. 1b) represent 3/21 ≈ 14 % of the original information, and five channels (Fig. 1c) 5/21 ≈ 24 % of the original information.

**Fig. 2:**
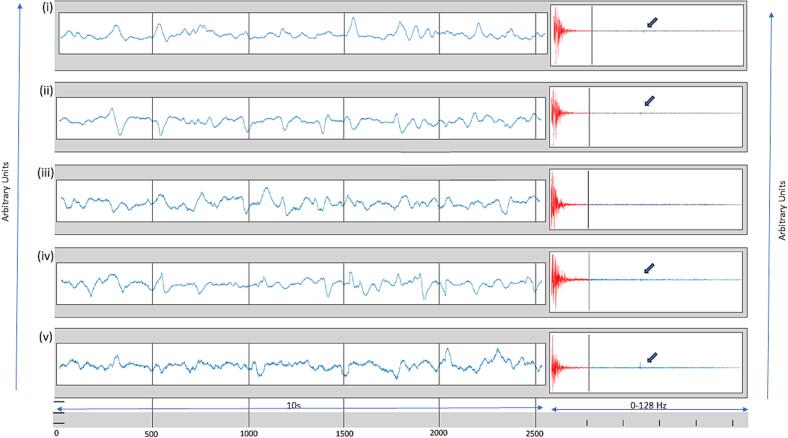

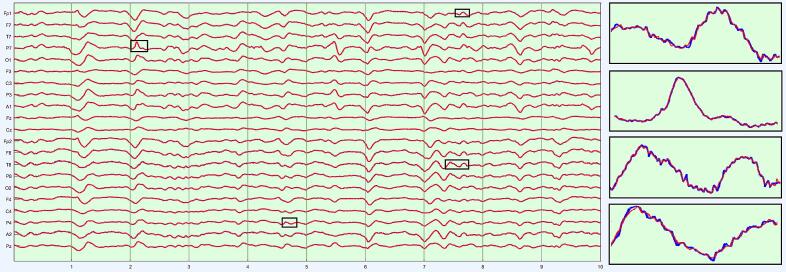
A) the five largest singular vectors extracted with svd of the 10-s EEG page of Fig. 2a, that in linear combination yielded the reduced EEG of Fig. 2c. The DCT of each singular vector is shown in the inset. Virtually all the power of DCT is observed in the lower frequencies, allowing for the top 20 % of coefficients to be those to the left of the vertical line. A small DCT spike (arrows) at 60 Hz represents line noise. b) Reconstruction of the SVD-reduced EEG of Fig. 1c. The first reconstruction (blue) reproduces that with the largest 5 singular vectors. The second reconstruction (red) derives from the largest 20 % DCT coefficients. The reconstructions are grossly visually identical. Magnification of the boxed areas (insets) show fine differences, the DCT reconstruction being a smoothed (low pass filtered) version of the SVD reconstruction in this example. (For interpretation of the references to colour in this figure legend, the reader is referred to the web version of this article.)

A second property of encephalopathic patterns is their limited repertoire of frequency content. Each discrete frequency in the EEG spectrum is a deterministic oscillation with high autocorrelation (thus, temporal redundancy). For encephalopathic EEG the oscillatory (spectral) content is sparse, due to the signal’s limited time-dependent repertoire, and the EEG well approximated by a small set of spectral coefficients. The discrete cosine transform is precisely a technique for characterizing a real signal by its spectral components, and Fig. 2a-b show its action on our data. The five SVD vectors of Fig. 1c are shown in Fig. 2a, with the insets showing their DCT spectra. The vectors added together in varying combinations yielded the full 21-channel EEG of Fig. 1c. Fig. 2b shows the 21-channel set reconstructed with largest 20 % the DCT components of the 5-set SVD. The two EEGs – DCT-naïve and DCT-filtered – are indistinguishable at normal viewing gains, though at fine scale (insets) the absence of fine rapid changes in the DCT-filtered time series is seen. The overall data reduction attained in this example by retaining five singular vectors, and then 25 % of their DCT components, was 24 % × 20 % = 4.8 %, equivalent to a compression ratio of <20.

**Fig. 3:**
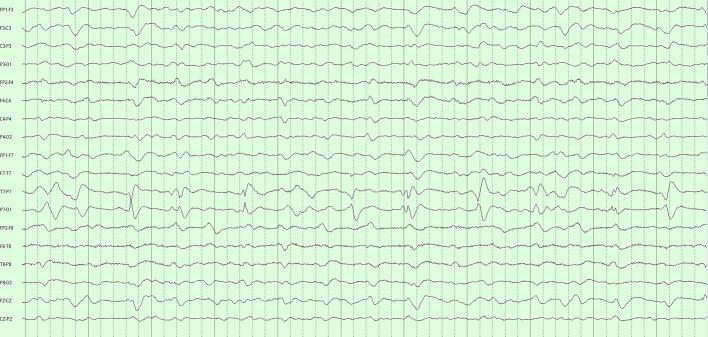

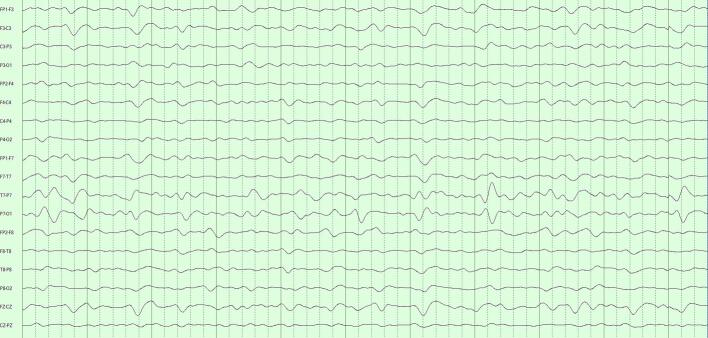

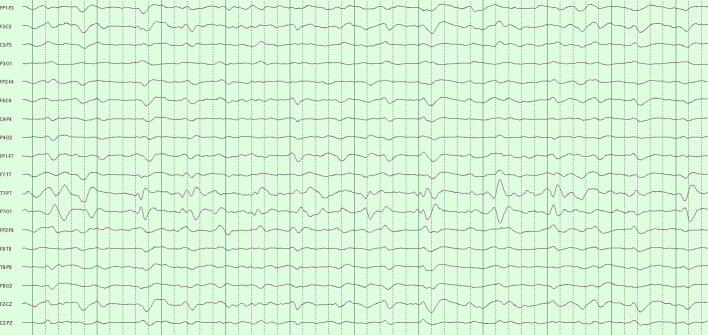
Comparison of COMP1 and COMP2 reconstructions in EEG with generalized slowing and left-lateralized periodic discharges (LPDs). a) Original EEG 10-s page, longitudinal bipolar montage, gain 7 μV/mm, passband 1–70 Hz. Generalized theta-delta background with superimposed ∼1/s left LPDs. b) COMP1 reconstruction adequately represents the background but LPDs appear blunted. c) COMP2 reconstruction more accurately reproduces the whole EEG.

Our decision for CR = 20 was motivated by an informal and approximate ‘one-day-reduced-to-one-hour’ ideal. Importantly, we found that COMP1 and COMP2, though achieving the same data compression, were not diagnostically equivalent. COMP1 compressed modestly with SVD and highly with DCT; COMP2 compressed equivalently with both techniques. The more balanced COMP2 formulation performed significantly better. These performances reflect how our algorithms interacted with the fundamental structure of LTM data. SVD is agnostic to the frequency content of the data, performing well with EEGs with diffuse features by picking up the low-rank structure of these appearances. Isolated events – whether slow or sharp – are poorly represented when SVD is truncated. The DCT is instead sensitive to the frequency content of the data. Polymorphic rhythms require an equivalent number of frequency components to be well-represented, with isolated high frequency events requiring the greatest number of DCT components of all. Fig. 3a–c illustrates the differential effects COMP1 and COMP2 reconstructions of an EEG with diffuse slowing and lateralized periodic discharge.

Scoring of original and reconstructed data by visual analysis was carried out by two blinded board-certified electroencephalographers. Statistical analysis assessed the differences in scores between original and reconstructed versions of the same EEG in comparison to the scorer’s intrinsic variability. We penalized differences (disagreements) along the scoring hierarchy. We observed no significant difference in the scores accorded to the original and Regime II reconstructed EEGs by the same reviewer, confirming our null hypothesis. Thus, the final arbiter of whether our methods were successful were how the data *looked*. It is in this respect – the human-centric nature of the performance ‘metric’ – that our study departs from metrics of performance applied in the engineering literature. Minimizing ‘error’ in clinical neurophysiology can be specific and idiosyncratic; visual diagnosis strives to capture the gestalt and is often robust to quite large distractors, such as ‘reading through’ muscle artefact to diagnose an underlying spike. However, the opposite situation – robustness of computer algorithms in the face of human fallibility – is just as true. Re-ordering a data matrix, for instance, would leave the SVD unchanged; reordering the sequence of channels would render an EEG quite uninterpretable to a viewer accustomed to standard montages. Similarly, time or voltage display rescaling confound visual interpretation but are inconsequential to computer algorithms.

A different question is what the SVD and DCT entities represented in themselves, if they somehow captured the biological ‘essence’ of the EEG. For DCT – a sum of cosines – these were simply the Berger bands. For SVD, the small number of singular vectors essentially meant an equivalently small number of spatiotemporal modes relevant to the generation of the relevant segment of EEG. In other words, the SVD was a form of source localization over the the head, with the individual ‘sources’ being single patterns of voltage distributions. An interesting question in this regard is the relation between the SVD sources and the ‘generators’ computed by conventional distributed source localization methods.

The current era of artificial intelligence and machine learning (AI/ML) may transform the practice of clinical neurophysiology with novel automated diagnostics (Lucas et al., 2024, Han et al., 2024, Abbasi and Goldenholz, 2019, Tveit et al., 2023), predictive modelling (Stirling et al., 2021, Sheikh and Jehi, 2024), data integration (Dasgupta et al., 2022, Duong et al., 2020) and data-mining (Craik et al., 2019). The success of AI/ML methods is as dependent on the curation of the appropriate data inputs (‘data-centric AI’) as the development and use of learning models (‘model-centric AI’) (Jarrahi et al., 2023). Our intention here was firmly data-centric: to dimension reduce the data to arrive at high-fidelity representations (as judged by visual review) for permanent archival. For longer recordings, the output of automated trend software (e.g. Persyst®^;^ Schomer and Hanafy, 2015, Scheuer et al., 2021) on the SVD-DCT reconstructed EEG is of future interest. Formal demonstration of trend software’s agnosticism to raw EEG or SVD-DCT reconstructions would provide additional clinical validation of our results.

## CRediT authorship contribution statement

**Giridhar P. Kalamangalam:** Conceptualization, Formal analysis, Writing – original draft, Writing – review & editing, Funding acquisition. **Subeikshanan Venkatesan:** Formal analysis, Project administration, Writing – original draft. **Maria-Jose Bruzzone:** Conceptualization, Writing – review & editing. **Yue Wang:** Writing – review & editing. **Carolina B. Maciel:** Conceptualization, Writing – review & editing. **Sotiris Mitropanopoulos:** Data curation, Writing – review & editing. **Jean Cibula:** Data curation, Writing – review & editing. **Kajal Patel:** Data curation, Project administration. **Abbas Babajani-Feremi:** Conceptualization, Formal analysis, Writing – review & editing.

## Declaration of competing interest

The authors declare that they have no known competing financial interests or personal relationships that could have appeared to influence the work reported in this paper.

## Appendix

SVD expresses the m×n data matrix A as the product

(1) $$A=U\Sigma V^{T}\text{.}$$

U is an orthogonal m×m matrix, Σ a diagonal m×n matrix containing singular values, which are all positive numbers arranged in a descending order of magnitude, and $V^{T}$ an orthogonal n×n matrix. For scalp EEG viewed in the usual way, m would be the list of head locations and n the successive data points. We subjected each 10-s epoch of 21-channel EEG – i.e., the successive m×n= 21×2560 number arrays – to SVD. Data reduction with SVD is understood from the equivalent formulation

(2) $$A=\sum_{i=1}^{m}\sigma_{m}u_{m}v_{m}^{T}$$

where the *u*’s and *v*’s are column vectors (of size 21×1 and 2560×1 for our data) that comprise the U and V matrices, *σ*’s are the singular values and each component outer product $u_{i}v_{i}^{T}$ is a rank-1 matrix. Due to the decreasing magnitudes of the singular values $\sigma_{1},\sigma_{2},\sigma_{3},$ etc., the major content of the matrix A might be captured by just the first few terms of the right side, i.e., by the approximation

(3) $$A\approx A_{r}=\sigma_{1}u_{1}v_{1}^{T}+\sigma_{2}u_{2}v_{2}^{T}+\sigma_{3}u_{3}v_{3}^{T}+\cdots \sigma_{r}u_{r}v_{r}^{T}\equiv U_{r}\Sigma_{r}V_{r}^{T}\text{,}\;r<m.$$

For our data, r in the range 5–10 captured 70–80 % of the variance in the original EEG. Using r=5, for instance, meant $A_{r}$ was the additive combination of five $uv^{T}$ products. With each product being specified by 21+2560+1=2582 numbers, the original matrix size 21×2560 was reduced to 5×2582, a saving of about 75%.

(ii) Discrete Cosine Transform (DCT): The set of vectors $v_{1},v_{2},\cdots v_{r}$ of the reduced matrix $A_{r}$ was next subjected to the DCT. The DCT is an algorithm that projects a sequence of data points on to a set of cosine functions of different frequencies. Several types of DCT are described. We adopted the commonly-used DCT-2 variant^7^ here, formally

(4) $$X\left(k\right)=\sqrt{\frac{2}{N}}\sum_{n=1}^{N}v_{i}\left(n\right)\frac{1}{\sqrt{1+\delta_{k1}}}\cos\left[\frac{\pi }{2N}\left(2n-1\right)\left(k-1\right)\right],k=1,2,\cdots N$$

where *δ_k_*_1_ is the Kronecker delta. The coefficients X(k) were the relative contributions of different cosine frequencies to the original signal (singular vector) v. The full set X was the same size as v, but to achieve data compression, only a specific number of the largest DCT coefficients were retained with the remaining coefficients set to zero. This truncation was similar in spirit to the process with SVD above, asserting that the majority of the information in vector *v* that was represented by its DCT *X* , were contained in the largest few coefficients of *X*. The retained DCT coefficients were then quantized to two bytes in preparation for run-length encoding below.

(iii) Run-length Encoding: In a final compression step, the quantized coefficients were subjected to run-length encoding (RLE), a technique that compresses sequences of zeros by representing them with a single zero value followed by a count, utilizing two bytes for the quantization bit number. For example, if a signal with 10 data points were transformed using DCT and only the two largest coefficients retained, the remaining eight would be set to zero. After quantization, the sequence might look like [55, −45, 0, 0, 0, 0, 0, 0, 0, 0]. RLE would compress the sequence by encoding the zeros efficiently as ([55, 1], [-45, 1], [0, 8]), where each pair represents a value and its count. For this example, the compression achieved by RLE would be the reduction of 10 numbers in the original sequence to six, i.e., (10 × 2 bytes) / (6 × 2 bytes) = 1.67. In practice, we needed a more nuanced approach, recognizing that while DCT allows precise control over the number of retained coefficients, the actual compression for RLE depends on the pattern of zeros in the sequence of coefficients. To achieve an exact desired compression, we used an iterative algorithm that retained a variable number of the largest coefficients, setting the rest to zero. The coefficients were quantized and then compressed using RLE. The overall CR (achieved by SVD and DCT together) was calculated for a given number of DCT coefficients, and the number of retained coefficients adjusted by Newton-Raphson iteration until the achieved CR matched a desired value.

(iv) Reconstruction: Inverse RLE expanded the compressed data back to the original sequence of quantized coefficients, which were then inverse quantized to restore their original range and precision. The inverse DCT was then applied to convert the frequency domain representation back to the time domain, thus reconstructing approximations $v_{1X},v_{2X},\cdots v_{\mathrm{rX}}$ to the original singular vectors $v_{1},v_{2},\cdots v_{r}$. The former set was assembled into the matrix $V_{\mathrm{rX}}$ and final data-reduced EEG reconstructed by multiplying out

(5) $$A_{R}=U_{r}\Sigma_{r}V_{\mathrm{rX}}^{T}\text{.}$$

Reconstructed 10-s epochs were concatenated into one-hour long reconstructions to partner their original versions. We experimented with changing the truncations of the SVD and DCT/RLE to achieve various degrees of compression, finally settling on a CR of 20. We evaluated two ways of achieving this CR as follows. Regime I: Individual SVD and DCT CR of 1.7 and 12 respectively (COMP1; 1.7 × 12 ≈ 20); and Regime II: CRs of SVD and DCT of 3.7 and 5.7, respectively (COMP2; 3.7 × 5.7 ≈ 20).
