# Supplementary figures and images for: COMPRESSIVE DATA STORAGE FOR LONG-TERM EEG: VALIDATION BY VISUAL ANALYSIS
Source: Clin Neurophysiol Pract. 2025 Aug 5;10:331–9. doi: 10.1016/j.cnp.2025.07.005 (PMC12344260; doi:10.1016/j.cnp.2025.07.005)

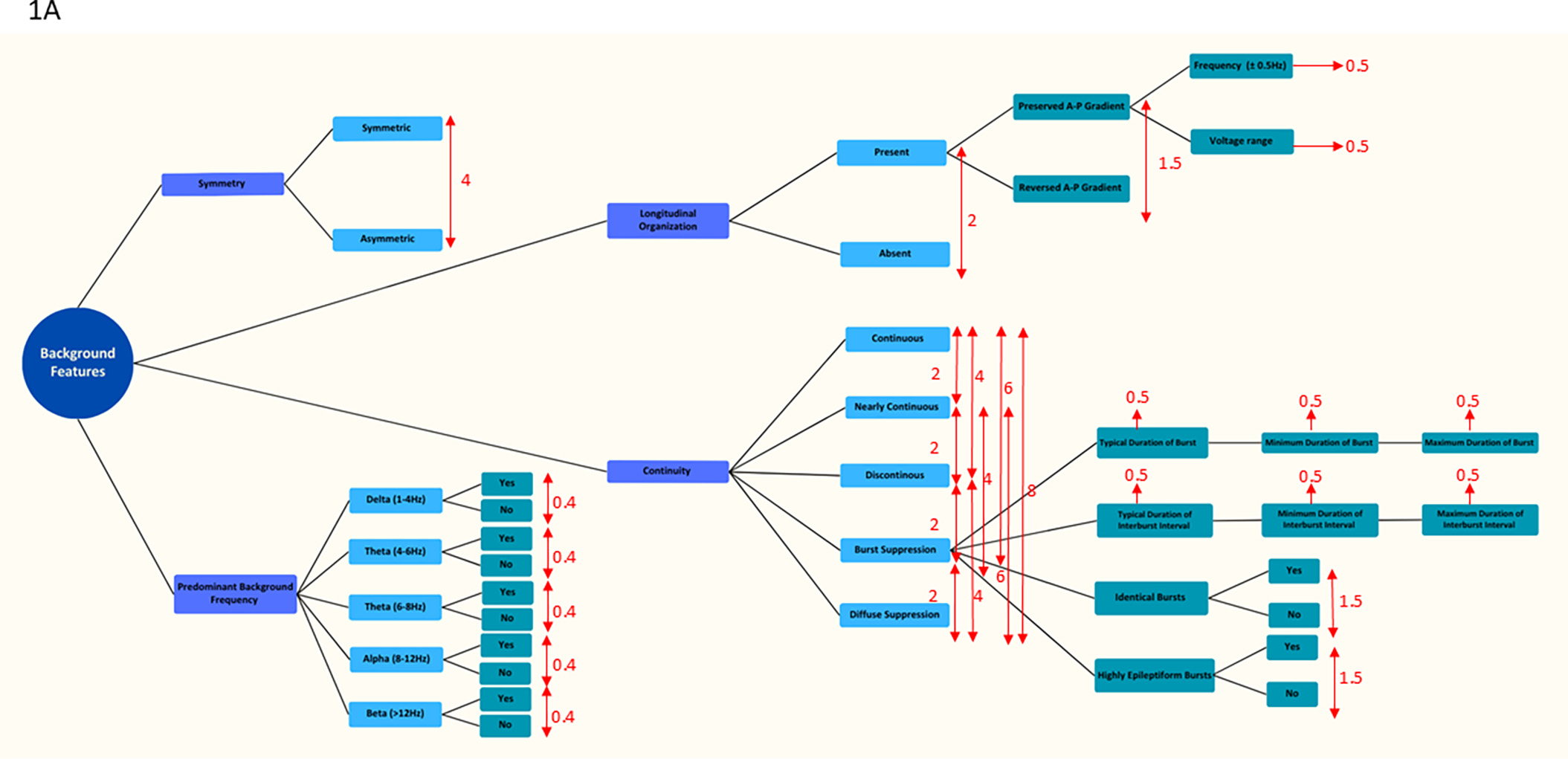

Supplement: Supplementary Fig. 1 [file mmc1.jpg]

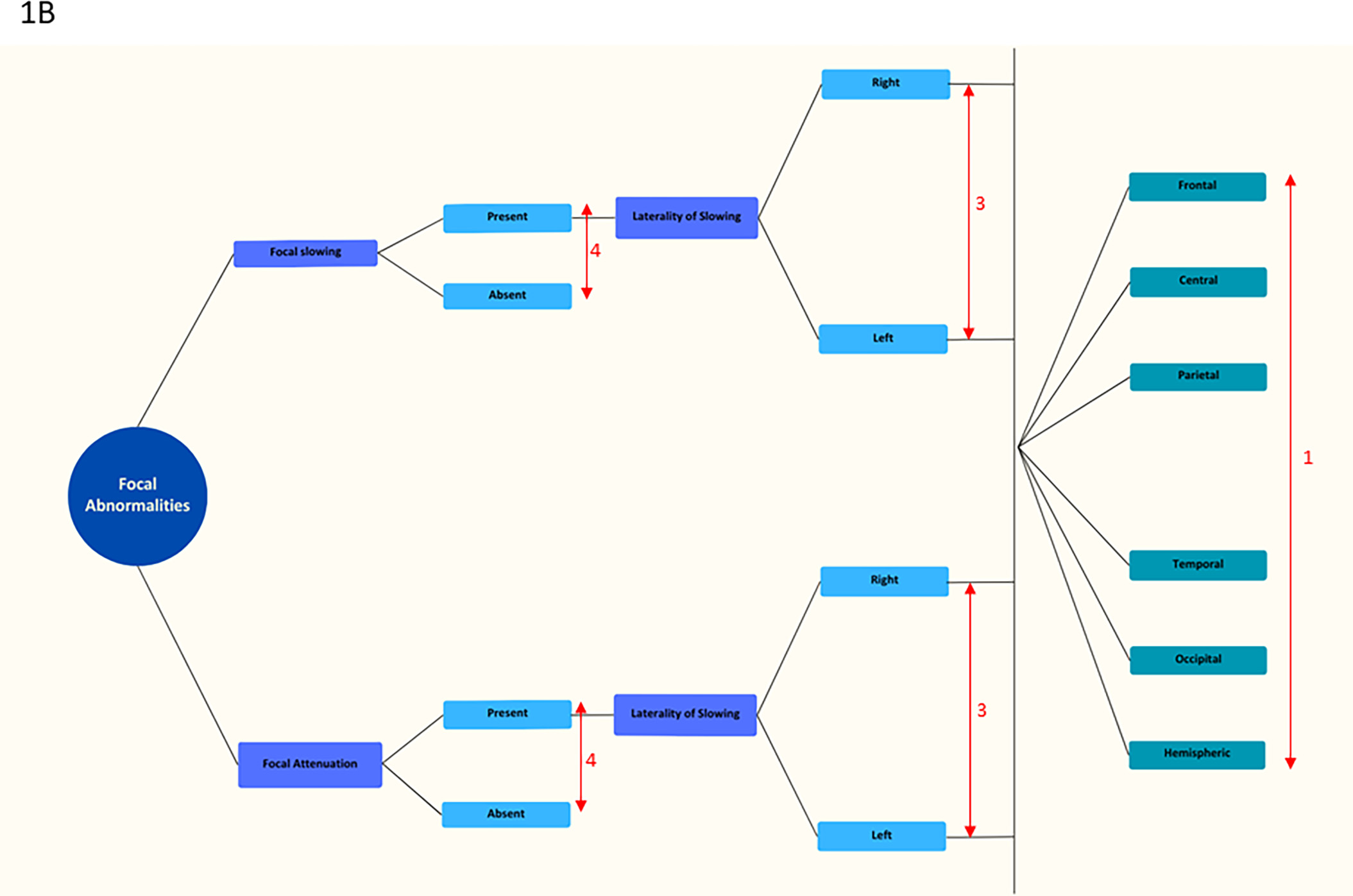

Supplement: Supplementary Fig. 2 [file mmc2.jpg]

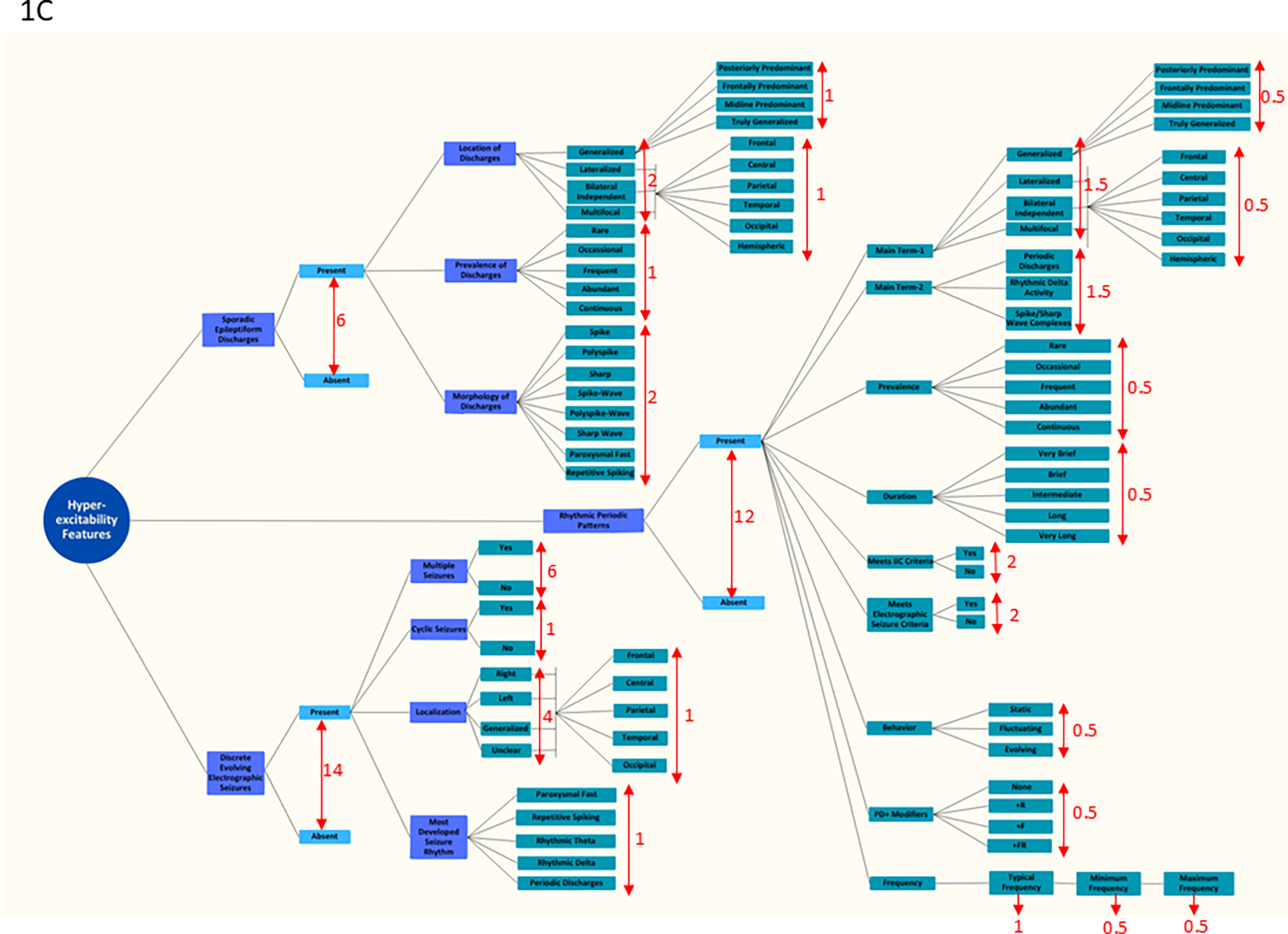

Supplement: Supplementary Fig. 3 [file mmc3.jpg]
