# Supplementary material for: COMPRESSIVE DATA STORAGE FOR LONG-TERM EEG: VALIDATION BY VISUAL ANALYSIS
Source: Clin Neurophysiol Pract. 2025 Aug 5;10:331–9. doi: 10.1016/j.cnp.2025.07.005 (PMC12344260; doi:10.1016/j.cnp.2025.07.005)
Supplement: Supplementary Data 4 [file mmc4.docx]

**Supplementary Figure captions**

A-C) Scoring scheme for visual review of EEG, adopted from the 2021 ACNS guidelines. Findings were classified along three main axes (a) background features, (b) focal findings, and (c) hyperexcitable features, each qualified further in the branching structure shown. Reviewers scored items following a left-to-right progression, either as a binary response (i.e., seizures: 1 - present and 0 - absent), grading on a nominal scale (e.g., localization of the seizure onset: 1 – right, 2 – left, 3 – generalized, or 4 - unclear), or a number (e.g. typical frequency of a rhythmic pattern). These responses did not directly enter statistical analysis. Rather, it was the degree of agreement or disagreement between scores accorded to different versions of the same EEG (ORIG1, ORGI2, COMP1 and CPOMP2; see text) that was of interest. These were analyzed with accorded penalties shown by the vertical red arrows. Disagreements further down the branches were less significant and carried less penalty than those closer to the main stems of the scoring tree.
